# Population PK/PD Model of Homocysteine Concentrations after High-Dose Methotrexate Treatment in Patients with Acute Lymphoblastic Leukemia

**DOI:** 10.1371/journal.pone.0046015

**Published:** 2012-09-25

**Authors:** Hauke Rühs, Achim Becker, Anne Drescher, John C. Panetta, Ching-Hon Pui, Mary V. Relling, Ulrich Jaehde

**Affiliations:** 1 Department of Clinical Pharmacy, Institute of Pharmacy, University of Bonn, Germany; 2 Department of Pharmaceutical Sciences, St. Jude Children’s Research Hospital, Memphis, Tennessee, United States of America; 3 Department of Oncology, St. Jude Children’s Research Hospital, Memphis, Tennessee, United States of America; West Virginia University School of Medicine, United States of America

## Abstract

Elevated homocysteine concentrations have been associated with methotrexate-induced neurotoxicity. Based on methotrexate and homocysteine plasma concentrations of 494 children with acute lymphoblastic leukemia treated with high-dose methotrexate in the TOTAL XV study, a pharmacokinetic/pharmacodynamic (PK/PD) model was built with NONMEM. Several compartment and indirect response models were investigated. The pharmacokinetic disposition of methotrexate was best described by a two-compartment model. Homocysteine concentrations were included by an indirect response model where methotrexate inhibition of the homocysteine elimination rate was described by an E_max_ model. The homocysteine baseline level was found to be age-dependent. Simulations revealed that folinate rescue therapy does not affect peak concentrations of homocysteine but leads to a modestly reduced homocysteine exposure. In conclusion, our PK/PD model describes the increase of methotrexate-induced HCY concentrations with satisfactory precision and can be applied to assess the effect of folinate regimens on the HCY concentration-time course.

## Introduction

Methotrexate (MTX) is a folate analogue which has been used for many years in the treatment of solid tumors [1], [2] and hematological malignancies, such as acute lymphoblastic leukemia (ALL) [3]–[6]. High-dose MTX (HDMTX) therapy is associated with severe adverse events including neurotoxicity, with acute events such as somnolence, confusion, fatigue, disorientation, seizures, headache and dizziness occurring during or within hours of administration [7], [8]. Subacute neurotoxic symptoms such as hemiparesis, ataxia, speech disorders, seizures, confusion and affective disturbances may occur within days to weeks after HDMTX exposure [8], [9]. In a clinical trial with HDMTX and folinate rescue 15% of the patients developed transient neurotoxic symptoms [10]. Moreover, leukoencephalopathy characterized by cerebral white matter changes has been associated with HDMTX treatment. The incidence of this chronic neurotoxicity is less than 2% following administration of intravenous HDMTX [8], [11].

**Figure 1:**
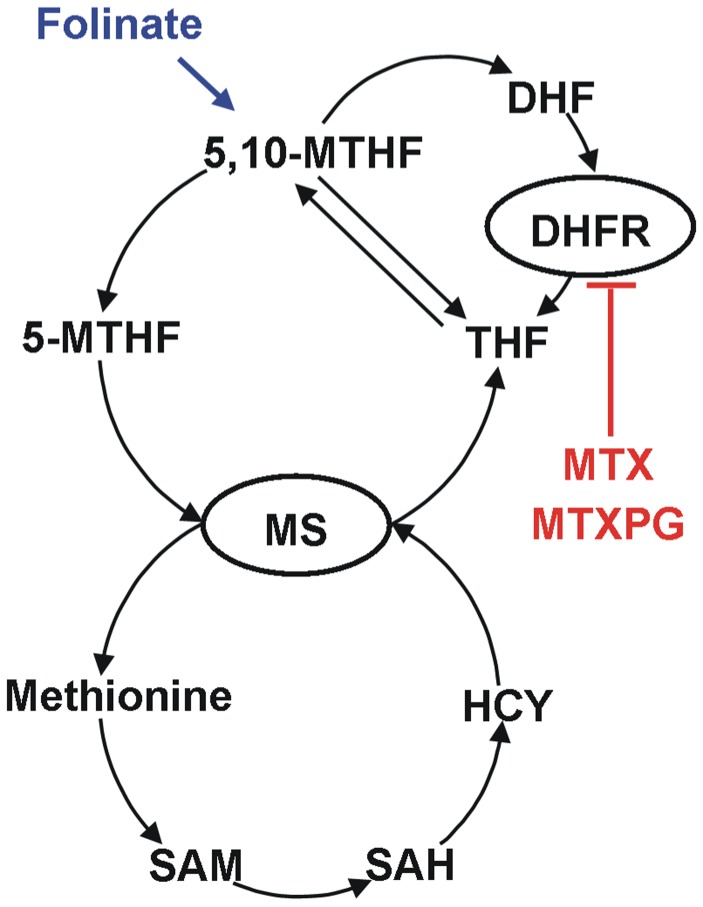
Scheme of the folate-homocysteine pathway (MTX = methotrexate; MTXPG = MTX polyglutamates; DHFR = dihydrofolate reductase; DHF = dihydrofolate; THF = tetrahydrofolate; 5-MTHF = 5-methyl-THF; 5,10-MTHF = 5,10-methylene-THF; HCY = homocysteine; MS = methionine synthase; SAM = S-adenosylmethionine; SAH = S-adenosylhomocysteine).

Methotrexate has been postulated to cause neurotoxicity by affecting the metabolism of the neurotoxic amino acid homocysteine (HCY). MTX and its polyglutamated forms (MTXPG) are inhibitors of dihydrofolate reductase (DHFR) resulting in a cellular depletion of tetrahydrofolates (THF). 5-methyl-THF (5-MTHF) and 5,10-methylene-THF (5,10-MTHF) are essential for the conversion of HCY to methionine by the enzyme methionine synthase (MS). Thus, the inhibition of the DHFR by MTX leads to accumulation of HCY and depletion of S-adenosylmethionine (SAM) [12]–[15]. There are indications that these alterations contribute to the observed neurotoxic symptoms [16]–[20]. Furthermore, a possible direct effect of MTX on the central nervous system (CNS) has been discussed [8]. The administration of folinate (Leucovorin®) after HDMTX therapy reduces the occurrence of neurotoxic events [21] because the intracellular pool of reduced folates is replenished and the effect of MTX is antagonized (see Figure 1).

Although there are various publications linking HCY with MTX toxicity, the number of patients and available samples was mostly too small to describe this relationship by means of a population pharmacokinetic/pharmacodynamic (PK/PD) model. In this study, the concentration-time course of HCY after the administration of HDMTX was monitored in a large front-line study, Total XV, for children with newly diagnosed ALL [22]. The aim of this investigation was to build a population PK/PD model in order to allow simulations of HCY concentration-time courses based on the dosage regimen of MTX and characteristics of individual patients. The model was applied to assess the potential effects of post-MTX folinate rescue on HCY exposure.

## Results

### Patient Characteristics

Of the 498 evaluable patients (1 to 18 years of age), 408 patients were enrolled at St. Jude Children’s Research Hospital and 90 patients at Cook Children’s Medical Center. For this investigation 3 patients were excluded due to missing MTX concentrations and one because of implausible MTX plasma concentrations. The main demographic characteristics of the remaining 494 patients are listed in Table 1.

**Table 1:** Patients characteristics.

		Index dataset331 patients1,492 courses		Evaluation dataset163 patients751 courses	
		Median	Range	Median	Range
MTX Dose [mg]	WIN	800	398–2983	785	444–2126
	CONS (LR)	2049	274–7187	1999	240–6293
	CONS (SHR)	4924	412–15524	4594	1261–14521
MTX Dose per BSA [mg/m^2^]	WIN	1000	847–1096	1003	946–1160
	CONS (LR)	2653	492–4610	2635	491–5626
	CONS (SHR)	4654	496–8580	4688	1797–8094
Serum creatinine [mg/dL]		0.4	0.1–1.2	0.4	0.1–1.1
BSA [m^2^]		0.83	0.40–2.97	0.80	0.43–2.21
Height [cm]		114.1	68.1–192.6	109.2	72.5–198.9
Weight [kg]		22.0	7.8–160.1	19.6	8.8–95.7
Age [years]		5.42	1.03–18.85	4.95	1.02–18.73
Gender (male/female)		189 (57.1%)/142 (42.9%)		87 (54.0%)/74 (46.0%)	
Risk group (LR/SHR)		175 (52.9%)/156 (47.1%)		88 (54.0%)/75 (46.0%)	

WIN = window phase; CONS = consolidation phase LR = low risk; SHR = standard/high risk.

For these 494 patients, 6722 MTX plasma concentrations were determined. From this population 2567 HCY plasma concentrations were determined in 414 patients before, during and after MTX administration, just immediately before folinate rescue therapy was given. The dataset was divided into an index and an evaluation dataset with 331 and 163 patients, respectively (see Table 1).

### Pharmacokinetics

**Figure 2:**
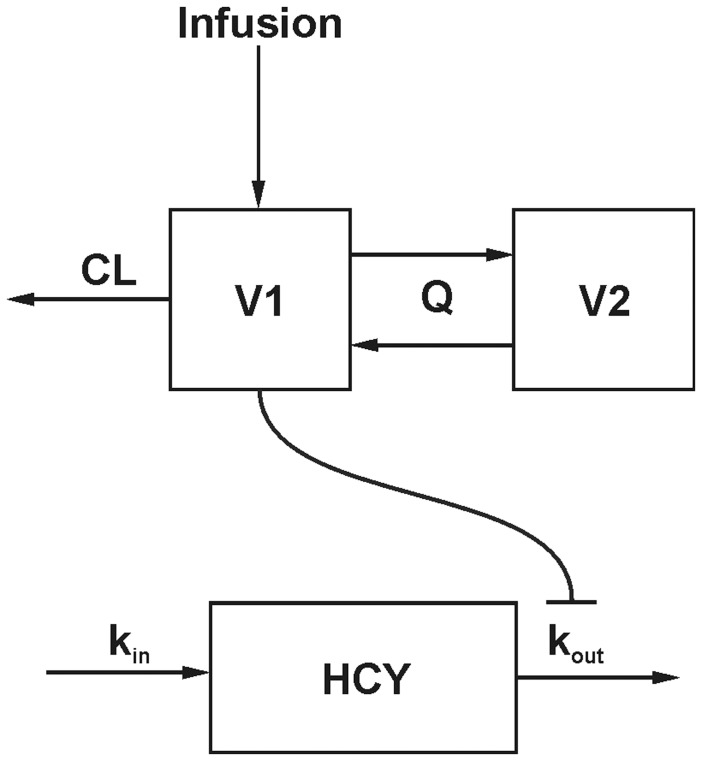
Scheme of the PK/PD model (CL = total body clearance; Q = intercompartmental clearance; V1 = volume of the central compartment; V2 = volume of the peripheral compartment; HCY = homocysteine; k_in_ = HCY formation rate constant; k_out_ = **HCY elimination rate constant).**

The best fit for the MTX concentrations was achieved by assuming a two-compartment model (see Figure 2). The structural parameters of this model were total body clearance (CL), volume of distribution of the central compartment (V1), intercompartmental clearance between central and peripheral compartment (Q) and volume of distribution of the peripheral compartment (V2). All parameters were scaled by BSA. Interindividual variability (IIV) was estimated by assuming with a log-normal distribution of the parameters CL, V1 and Q. All estimated parameters are shown in Table 2. An exponential error model best described the residual error of the model. An interoccasion variability (IOV) was included to describe variations of CL within one patient between the window administration and the four HDMTX administrations. IOV reduced the objective function value (OFV) by 250.58 from −3997.57 to −4248.15. Serum creatinine (C_CR_) was found as a covariate on CL in a forward inclusion procedure and significance could be confirmed by a backward exclusion step (p<0.001). C_CR_ was adjusted for age and gender of the patient (C_CR,adj_) to account for maturation-dependent differences [23]:
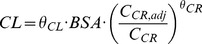
(1)


**Table 2:** Pharmacokinetic and pharmacodynamic model parameters.

	ModelEstimates(CI_90%_)	Interindividual variability(CI_90%_) [%]	InteroccasionVariability(CI_90%_) [%]
**PK Parameters**			
θ_CL_ [L/h/m^2^]	6.68 (6.56–6.82)	14.53 (12.77–15.81)	17.15 (15.81–18.44)
θ_V1_ [L/m^2^]	18.6 (17.69–19.57)	18.03 (14.97–22.58)	–
θ_Q_ [L/h/m^2^]	0.161 (0.147–0.177)	36.61 (28.50–39.62)	–
θ_V2_ [L/m^2^]	3.09 (2.81–3.53)	–	–
θ_CR_	0.314 (0.251–0.375)	–	–
Exponential Error	0.235 (0.220–0.257)	–	–
**PD Parameters**			
θ_BL_ [µM]	4.88 (4.74–5.02)	18.95 (15.52–22.00)	23.83 (20.69–27.15)
θ_kout_ [h^−1^]	0.027 (0.026–0.030)	33.23 (23.96–37.47)	–
θ_Emax_	1 (fixed)	–	–
θ_EC50_ [µM]	0.648 (0.483–0.965)	53.63 (13.08–142.43)	–
θ_BL,AGE_ [µM/year]	0.116 (0.091–0.150)	–	–
Exponential error	0.165 (0.146–0.177)	–	–
Additive error [µM]	0.911 (0.783–1.046)	–	–

CI_90%_ = 90% confidence interval.

(CI_90%_ by bootstrapping).

**Figure 3:**
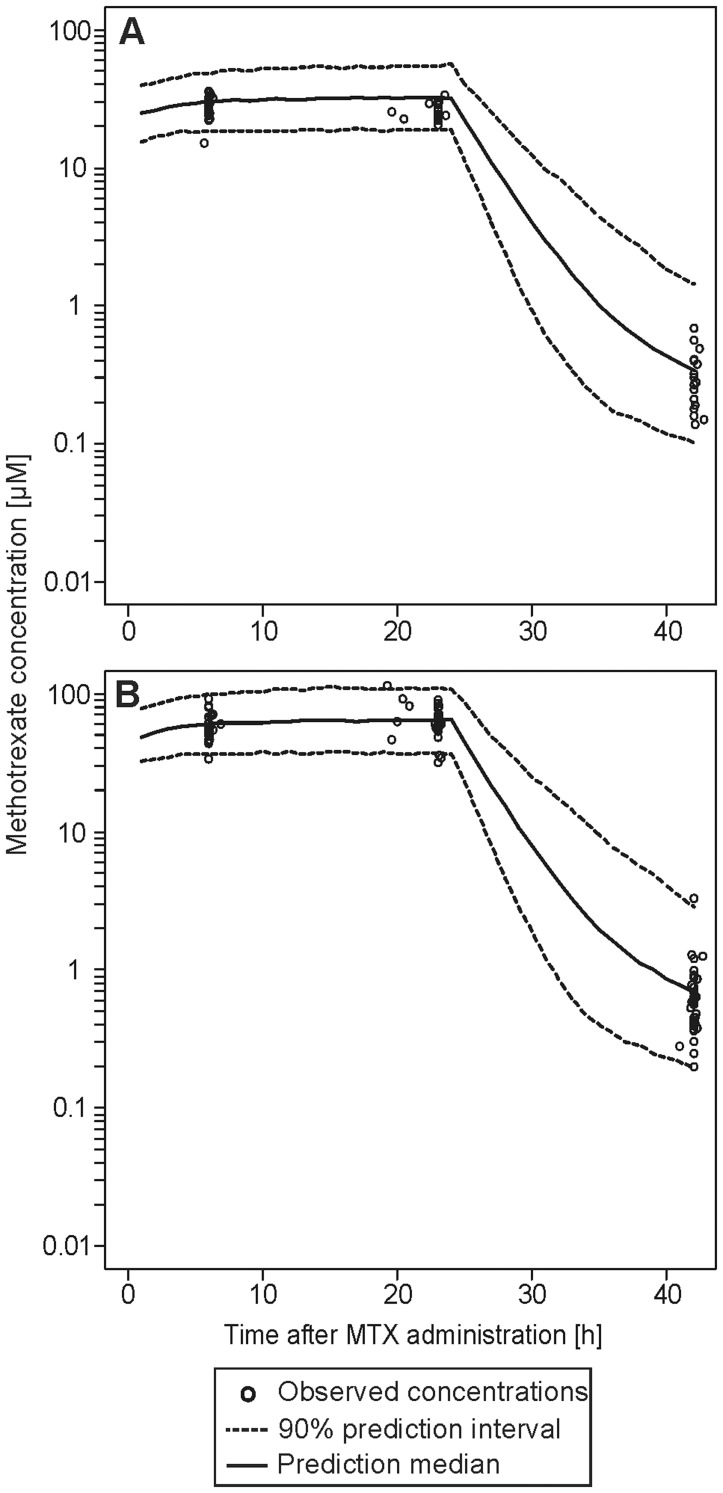
Visual predictive check of the pharmacokinetic model for all patients of the evaluation dataset receiving a 24 h MTX infusion with a dose of 2500±20 mg/m^2^ (A) and 5000±20 mg/m^2^ (B), respectively. The black circles represent the measured methotrexate concentrations. The solid line represents the median predicted concentration, the dashed lines represent the 90% prediction interval of 10000 simulations.

The PK model then was evaluated visually by a visual predictive check (VPC) plot of the evaluation dataset (see Figure 3).

### Pharmacodynamics

**Figure 4:**
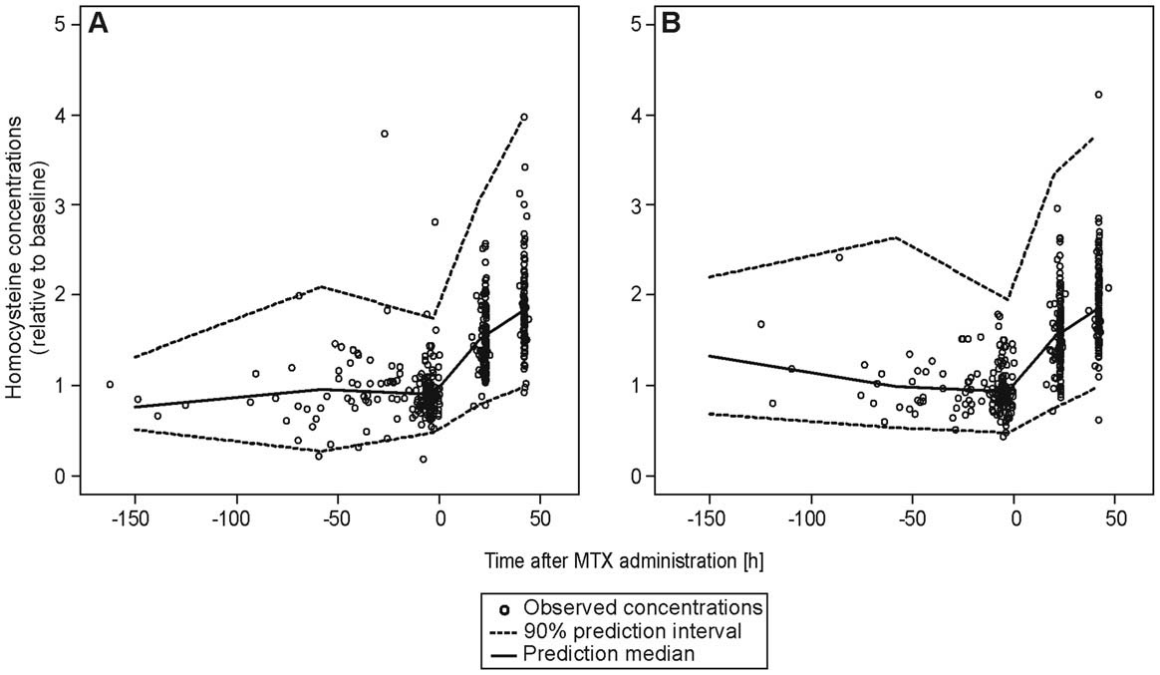
Visual predictive check of the pharmacodynamic model for low-risk (A) and standard/high-risk (B) patients from the evaluation dataset. The black circles represent the measured homocysteine concentrations relative to baseline. The solid line represents the median predicted concentration, the dashed lines represent the 90% prediction interval of 1000 simulations.

An indirect response model was used to describe the HCY concentrations. An E_max_ model was used to link the PK and PD model. The structural parameters HCY baseline concentration (HCY_BL_), HCY elimination rate constant (k_out_) and EC_50_ were successfully estimated. E_max_ was fixed to a value of 1, as any attempt to estimate this parameter did not improve the model. The IIV was estimated for HCY_BL_, k_out_ and EC_50_ assuming a log-normal distribution. Additionally, the inclusion of IOV on HCY_BL_ significantly improved the model by reducing the OFV 250.6 units (p<0.001), and also reduced IIV_CL_ and the residual error. The residual error was established for the HCY data by a model using a combination of an additive error of 0.911 µM, which was dominant for lower concentrations and an exponential error of 0.165 for higher concentrations. Age was the only significant covariate; baseline HCY levels significantly increased with respect to age (p<0.001). To assess the predictability of the model, a VPC was performed on the HCY change from baseline concentration (see Figure 4).

### Effect of Folinate Rescue

**Figure 5:**
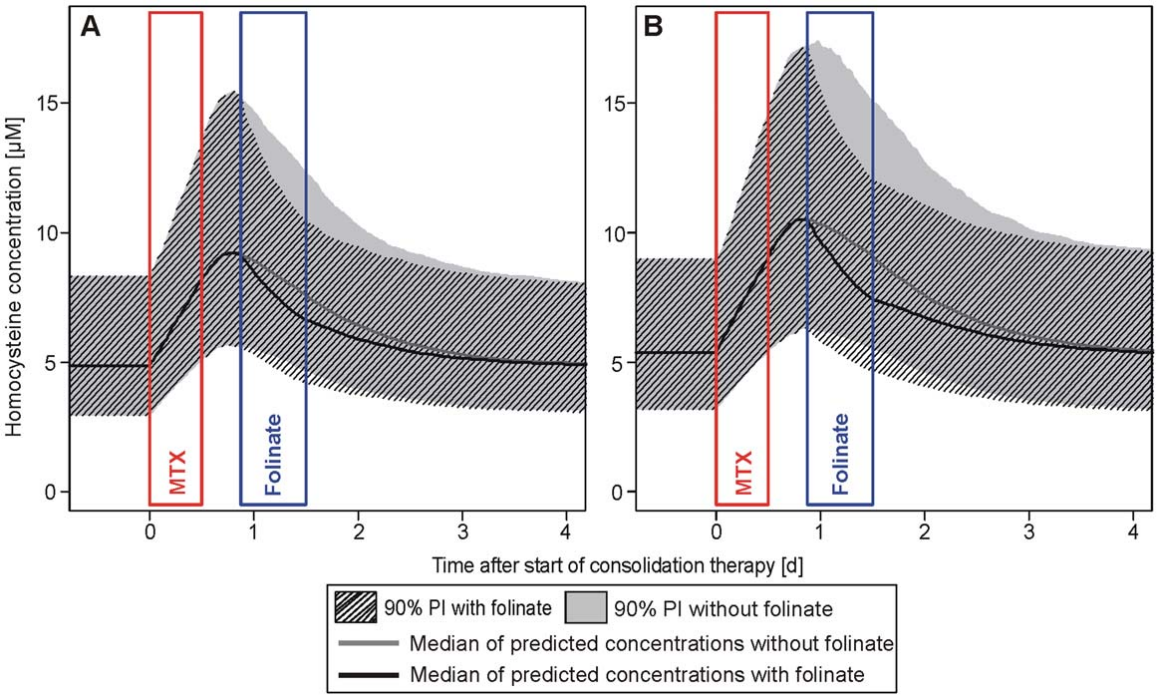
Homocysteine concentration-time courses with and without co-administration of folinate. Excerpt of 1000 per-protocol simulations each of the low-risk (A) and standard/high-risk (B) study population. The solid grey line represents the median of the simulations without folinate with the 90% prediction interval (PI) as solid grey area, the solid black line represents the median of the simulations under influence of folinate with the 90% PI as shaded area.

The 90% prediction intervals (PI), based on the bootstrap simulations, were calculated with and without folinate influence for the low-risk (LR) and standard/high-risk (SHR) subgroups (see Figure 5). The simulations show that HCY concentrations continued to increase after the end of the 24 hour MTX infusion with or without folinate rescue in both subgroup simulations. The median t_max_ of HCY was reached at 15 hours after the end of MTX infusion and 3 hours before the onset of the folinate rescue in the LR group. In the SHR simulation group the median t_max_ was reached at 18 hours after the end of MTX infusion under folinate influence and at 19.3 hours without folinate influence. HCY concentrations declined faster in the presence of folinate, as at the end of folinate treatment the median HCY concentrations were significantly (p<0.001) lower in both subgroups (see Figure 5). In the LR subgroup the median C_max_ of HCY was estimated to be 9.37 µM (90% PI: 5.73–15.77) without and 9.33 µM (5.69–15.77) with folinate (not significant), the median HCY AUC over baseline concentration decreased by 15.9% from 1615 µM•h (90% PI: 951–2765) to 1358 µM•h (90% PI: 843–2180) under the influence of folinate (p<0.001). In the SHR subgroup the median C_max_ of HCY was estimated to be 10.70 µM (90% PI: 6.37–17.84) without and 10.56 µM (6.31–17.41) with folinate which was not significantly different (p = 0.34), whereas the median HCY AUC over baseline concentration decreased by 21.5% from 2107 µM•h (90% PI: 1207–3510) to 1653 µM•h (90% PI: 994–2593) under the influence of folinate (p<0.001). Both simulated AUC values over baseline in the LR subgroup were significantly lower when compared to those from the SHR subgroup (p<0.001, each).

## Discussion

In the literature there are several hints that HDMTX administration affects HCY exposure. Patients treated with MTX exhibited significantly increased CSF concentrations of HCY compared to a control group [16]. An increase of HCY concentrations has also been shown in plasma [13], [24]. There was a weak positive correlation between the MTX plasma concentrations at 23 hours and the HCY concentration at 44 hour after the start of MTX infusion [18]. In order to improve the description of this phenomenon and to allow simulations of HCY concentration-time courses we conducted a population PK/PD analysis linking HCY and MTX plasma concentrations using an indirect response model.

Similar to previously published population PK models for MTX [25]–[29], a two-compartment model was used to describe the MTX concentrations. When normalized for BSA, the PK parameters estimated in our study lie within the range of those reported in the literature. Serum creatinine concentration was a significant covariate on CL which is also consistent with several other studies [25]–[27]. The validity of population PK parameters estimated (index dataset) was confirmed with an independent group of patients (evaluation dataset).

The formation of HCY by several enzymes and its demethylation via MS to methionine are described in this model by the HCY formation rate constant (k_in_) and elimination rate constant (k_out_), respectively. As only HCY concentrations were available at baseline or while still increasing around 23 and 42 hours after the start of MTX infusion, we had a lack of information when HCY concentrations declined again. Therefore we had to fix a parameter in the model. We chose E_max_, as any attempt of estimating this parameter led to values approximating 1. Thus, fixing E_max_ to 1 did not significantly corrupt the model in terms of the OFV.

The continuous formation and elimination of HCY leads to a steady-state baseline concentration of HCY (HCY_BL_) which was individually estimated. In a similar manner to other studies, we established a relationship between HCY_BL_ and age [30], [31]. While we did not include a circadian baseline level of HCY [32], we estimated an IOV on HCY_BL_ to contribute for intra-individual variability. The depletion of reduced folates due to the reversible inhibition of DHFR by MTX and its intracellularly formed polyglutamates [33] leads to an accumulation of HCY. This was described in our model by an inhibition of k_out_. In low- and standard/high-risk patients, HCY elimination rate (k_out_) was reduced to 1.93 and 0.99% of the baseline k_out_, respectively. Thus, conversion of HCY was inhibited almost completely in both risk groups. Hence, differences in maximum HCY plasma concentrations of those two subpopulations could not been shown. Any further increase in HCY concentrations was subsequently covered by the folinate rescue therapy antagonizing the inhibition of HCY degradation. This is also consistent with earlier findings presented in literature, as elevated HCY concentrations were observed after the start of MTX therapy until the folinate rescue was started [13], [24]. Nevertheless, the data from the evaluation dataset could be adequately described by the model (see Figure 4).

The PK/PD model was also used to simulate 1,000 HCY concentration-time courses during HDMTX therapy for the LR and SHR subgroup. The continued increase of the HCY concentrations after the end of the MTX infusion was due to the remaining systemic MTX. The steeper decrease of HCY concentrations under folinate rescue is plausible, as the pool of reduced folates is replenished by folinate and the enzyme MS can again convert more HCY to methionine. As a consequence, the AUC over baseline of HCY is significantly reduced. In the simulation setting the infusion ends exactly 24 hours after the start of infusion and the folinate rescue therapy is started exactly 18 hours later. As in the LR group t_max_ is reached before the folinate onset, no difference in the median t_max_ is observed with or without folinate. In contrast, in the SHR group the t_max_ without folinate is reached after the start of folinate treatment so that under folinate influence t_max_ is reached earlier in this subgroup. Nevertheless, no significant difference in the HCY C_max_ values was found between simulations with and without folinate influence.

In conclusion, our PK/PD model quantitatively describes the increase of HCY concentrations after HDMTX treatment. The model can be extended by including genetic determinants of MTX and HCY concentrations. As HCY is assumed to play a role in HDMTX-induced neurotoxicity, the model could also help to link MTX and HCY concentrations to clinical neurotoxicity, e.g. by predicting the severity of neurotoxic symptoms by individual HCY exposure and/or PD parameter estimates.

## Patients and Methods

### Patients

Details of the study design and the primary clinical results are reported elsewhere [22]. In brief, eligible patients with newly diagnosed ALL were enrolled on the Total Therapy XV study at St. Jude Children’s Research Hospital and at Cook Children’s Medical Center from June 2000 to October 2007. The protocol was approved by the St. Jude Children’s Research Hospital Institutional Review Board and the Cook Children’s Medical Center Institutional Review Board. Written informed consent was obtained from the patients who were 18 years old and, in the case of younger patients, from the parents or guardians, with assent from the patients, as appropriate.

Risk classification was based on the characteristics of the patients and treatment response. Patients with B-cell–precursor ALL who were between 1 and 10 years of age and had a leukocyte count of less than 50×10^9^ per liter, a DNA index (the ratio of DNA content in leukemic cells to that in normal diploid G0/G1 cells) of 1.16 or more, or the presence of translocation t(12;21)(*ETV6-RUNX1*) were provisionally classified as having low-risk ALL. Patients with the t(9;22)(*BCR-ABL1*) were considered to have high-risk ALL, and the remaining patients were initially classified as having standard-risk (intermediate-risk) ALL. The final risk status was determined on the basis of the level of minimal residual disease. Any patient with a level of minimal residual disease of 1% or more in the bone marrow aspirate on day 19 of remission induction or 0.10 to 0.99% minimal residual disease after completion of 6 weeks of induction therapy was considered to have standard-risk ALL. A level of minimal residual disease of 1% or more after completion of induction therapy indicated high-risk ALL.

### Treatment

Patients were randomly assigned to receive initial treatment with MTX over a period of 4 or 24 hours (window therapy). Those randomized to receive short infusions (4 hours) received 1 g/m^2^ in 100 ml dextrose (5%) solution. Patients who were randomized to receive long infusions received 200 mg/m^2^ MTX as an IV bolus injection, followed by 800 mg/m^2^ MTX in 250 ml dextrose (5%) solution over 24 hours. Folinate rescue for both infusion durations was initiated between 42 and 48 hours after the start of infusion (following the last blood sample), with an initial dose of 50 mg/m^2^ and 7 consecutive doses of 15 mg/m^2^ every 6 hours.

Four days later, remission-induction therapy was initiated with prednisone, vincristine, daunorubicin, and asparaginase. Patients with a level of minimal residual disease of 1% or more on day 19 received three additional doses of asparaginase. Subsequent induction therapy consisted of cyclophosphamide, mercaptopurine, and cytarabine.

On hematopoietic recovery (between days 43 and 46), the minimal residual disease was assessed, and consolidation therapy with HDMTX (every other week for 4 doses) and daily mercaptopurine for 8 weeks was started. Individual doses of HDMTX were calculated based on the patients’ CL from previous MTX administrations. The doses were adjusted to achieve a MTX steady-state plasma concentration (Cp_ss_) of 33 µM (estimated median Cp_ss_ for a 2500 mg/m^2^ dose) in the LR patients and 65 µM (estimated median Cp_ss_ for a 5000 mg/m^2^ dose) in the SHR patients, respectively. 10% of the individualized doses were administered over 1 hour as a loading dose and the remaining 90% subsequently over 23 hours. At 42 hours after start of MTX infusion, folinate rescue therapy was started. SHR patients received 15 mg/m^2^ and LR patients 10 mg/m^2^ folinate, respectively. These doses were given 5 times every 6 hours. The dosage of folinate was increased in patients with MTX concentrations >1.0 µM at 42 hours and continued until the MTX concentration was less than 0.1 µM.

### Sampling and Assays

During window therapy, plasma MTX concentrations were measured at 4, 24 and 42 hours after start of infusion, whereas during consolidation therapy plasma MTX concentrations were assessed at 6, 23 and 42 hours. MTX concentrations were determined by a fluorescence polarization immunoassay (Abbott TDx®, Abbott Laboratories, North Chicago, IL) with previously described methods [34], [35]. HCY plasma concentrations were measured in plasma samples drawn before administration of MTX and at 23 and 42 hours after start of infusion during the first two consolidation phases. HCY plasma concentrations were determined using a commercially available fluorescence polarization immunoassay (FPIA) kit (IMx® System Homocysteine, Abbott Laboratories, North Chicago, IL). The within- and between-day precision of this assay ranged from 1.4 and 2.2% coefficient of variation (CV) and 3.7 and 5.2% CV, respectively.

### PK/PD Model Building

Model building was performed with data from 2/3 of the patients (index dataset). The remaining 1/3 were used to perform an external evaluation (evaluation dataset). The assignment to the respective datasets was done randomly using the software R version 2.12.0 [36]. For the PK/PD analysis a population approach by nonlinear mixed-effect modeling was applied, using the software NONMEM® version 7.1.2 [37]. The first-order conditional estimation (FOCE) method with interaction was used for parameter estimation. Model selection was based on significant changes in the OFV and visual inspection of goodness-of-fit (GOF) plots. Specifically, two hierarchical models differing by one degree of freedom were considered significantly different if the OFV (-2 log-likelihood function) decreased at least 3.84 units (p<0.05; based on the χ^2^-test). The GOF plots were created by the Xpose 4.3.0 package [38] in R version 2.12.0 [36].

The PD model was built sequentially based on the PK model, i.e. the individual parameter estimates from the PK model were fixed, then the individual PD model parameters were estimated. The PD model was described by an indirect-effect model [39], describing the MTX concentration-dependent inhibition of HCY elimination by an inverse E_max_ model (see Figure 2). In the absence of MTX, a constant baseline concentration of HCY was assumed. Under steady-state conditions the following equation describes the relationship between k_in_ and k_out_:
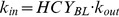
(2)where HCYBL and k_out_ were estimated by the model. Accumulation of HCY concentrations (C_HCY_) was assumed to be related to MTX concentrations (C_MTX_) and described by the following equation:
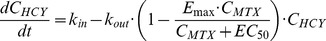
(3)where Emax is the remaining fraction to which kout will be reduced at the maximal effect of MTX and EC50 is the MTX concentration at which 50% of this maximal effect are reached. For each model, several error models were investigated (i.e. additional, proportional, exponential or combined error models).

The following covariates were available for investigating their potential influence on PK/PD parameters: gender, C_CR_, and age. Covariates were selected in the final population model by the forward-inclusion and backward-deletion approach. Covariates were included if their influence was plausible, variability on the pharmacokinetic parameter could be reduced, OFV could be significantly (p≤0.05) decreased and a significant (p≤0.005) increase in OFV occurred, when the covariate was excluded from the final model.

### Model Evaluation

The robustness and accuracy of the final model were evaluated with the index dataset by bootstrapping using the software Perl-speaks-NONMEM (PsN) [40], [41]. This procedure was performed 1000 times and led to non-parametric confidence intervals (CI) and mean values of all PK/PD parameters. Further a VPC was generated for evaluation of the final model. Specifically, using the evaluation dataset Monte Carlo simulations were generated. The resulting prediction intervals were compared visually to the original concentration-time points of the evaluation dataset.

### Simulations

The aim of the simulation studies was to investigate the influence of folinate rescue therapy on the HCY concentration-time profile. Stochastic simulations were carried out based on the MTX dose adaptation algorithm from the study protocol. The simulations were performed using R 2.12.0 [36]. To create a simulation population, 1000 patients were randomly drawn from the original population with replacement by using the bootstrapping method. Based on the individual NONMEM parameter estimates, 90% PI were calculated for the entire patient population with and without folinate co-administration. Folinate dosing was according to the protocol starting 44 hours after start of infusion in the window phase and 42 hours in the consolidation phase. It was assumed that the presence of folinate reversed the MTX effects on HCY completely, i.e. eq. 3 describing the HCY concentration-time course was simplified to.
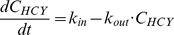
(4)during rescue therapy. A Wilcoxon rank sum test was used for statistical analysis. A p value less than 0.05 indicated statistical significance.
